# Outcomes of Patients Who Have Do Not Resuscitate Status prior to Being Admitted to an Intensive Care Unit

**DOI:** 10.1155/2016/1513946

**Published:** 2016-03-13

**Authors:** Debjit Saha, Carlos Moreno, Marc Csete, Elizabeth Kury Perez, Luigi Cubeddu, David Farcy, Steven Henry, Zachary Glazer, Lisa A. Moreno-Walton, Robert C. Goldszer

**Affiliations:** ^1^Mount Sinai Medical Center, Department of Internal Medicine, Miami Beach, FL 33140, USA; ^2^Health Professions Division, Nova Southeastern University, Miami Beach, FL 33140, USA; ^3^Mount Sinai Medical Center, Intensive Care Units, Miami Beach, FL 33140, USA; ^4^Information Technology Group, Mount Sinai Medical Center, Miami Beach, FL 33140, USA; ^5^Louisiana State University Health Sciences Center New Orleans, 1542 Tulane Avenue, New Orleans, LA 70112, USA

## Abstract

Admission of patients who have do not resuscitate (DNR) status to an intensive care unit (ICU) is potentially a misallocation of limited resources to patients who may neither need nor want intensive care. Yet, patients who have DNR status are often admitted to the ICU. This is a retrospective review of patients who had a valid DNR status at the time that they were admitted to an ICU in a single hospital over an eighteen-month period. Thirty-five patients met the criteria for inclusion in the study. The primary reasons for admission to the ICU were respiratory distress (54.2%) and sepsis (45.7%). Sixteen (45.7%) of the patients died, compared to a 5.4% mortality rate for all patients admitted to our ICU during this period (*p* < 0.001). APACHE II score was a significant predictor of mortality (18.5 ± 1.3 alive and 23.4 ± 1.4 dead; *p* = 0.038). Of the 19 patients discharged alive, 9 were discharged home, 5 to hospice, and 4 to a post-acute care facility.* Conclusions.* Patients who have DNR status and are admitted to the ICU have a higher mortality than other ICU patients. Those who survive have a high likelihood of being discharged to hospice or a post-acute care facility. The value of intensive intervention for these patients is not supported by these results. Only a minority of patients were seen by palliative care and chaplain teams, services which the literature supports as valuable for DNR patients. Our study supports the need for less expensive and less intensive but more appropriate resources for patients and families who have chosen DNR status.

## 1. Introduction

As the population ages and medical technology enables physicians to prolong the lives of patients with terminal diagnoses, DNR orders appear more frequently in patients' medical records. It is universally accepted for patients with DNR orders to not want intervention when signs of life cease, but there is considerable ambiguity about the level of treatment that is indicated for or desired by these patients when they present to the Emergency Department or to a physician's office with a treatable condition. When this condition is potentially life threatening and might better be treated with intensive intervention, these patients are often admitted to an ICU. The impact of caring for DNR patients in the ICU has not been fully evaluated [1]. Current literature addresses only 28-day mortality for patients admitted to ICU with DNR status (OR 3.64; CI 3.14–4.21, *p* < 0.001) [2]. Increasing our understanding of the impact of ICU care for DNR patients may result in increased patient and family satisfaction and physician and staff satisfaction and improved ICU utilization. There is a need for increased data on evidence based end of life care [3].

## 2. Methods

This is a retrospective chart analysis reviewed and approved by the Mount Sinai Medical Center (MSMC) Institutional Review Board. The MSMC electronic health record (EHR) was used to identify all patients who had a valid DNR status at the time that they were admitted from January 2013 to July 2014. Patients were admitted to one of four mixed medical-surgical ICUs with a total of 60 beds. All-cause mortality prior to discharge was calculated and compared to non-DNR hospital patient mortality. For the subset of DNR patients admitted to the ICU, charts were reviewed for admission diagnosis, APACHE II score, serum creatinine and lactate levels, endotracheal intubation, use of pressor therapy, hemodialysis, ICU length of stay, palliative care or chaplaincy consultation, and discharge status. Results are expressed as absolute values, percentages, and as means ± standard error of the mean (SEM). Statistical differences were calculated by Student's *t*-test and Fisher exact test. *p* values of ≤0.05 were considered significant.

## 3. Results

During the eighteen-month study period, DNR patients accounted for 2.2% (*n* = 686) of all hospital admissions and had a mortality rate of 36% (247/686), in comparison to hospital mortality rate of 1.5% excluding DNR patients (*p* < 0.001). There were 35 patients identified who had a valid DNR prior to admission to the ICU constituting 0.48% of all ICU admissions or 5.1% of all hospital DNR admissions. The mean age of DNR patients admitted to the ICU was 80.3 years (±2.1), and 57% were female. The mean serum creatinine was 1.6 mg/dL (±0.17), mean serum lactate level was 2.8 mmol/L (±0.5), and the mean APACHE score was 20.9 (±1.1).

Of the 35 patients in the study, 16 (45.7%; 95% CI = 29.2–62.2%) died (Table 1). Mortality for all patients who were DNR at admission or became DNR during hospitalization during the study period was 33.4% (*N* = 103/308; 95% CI = 28.2–39.1). Mortality for all patients that were admitted to the ICU during the study period and were not DNR was 4.95% (*N* = 270/5487; *p* < 0.001; 95% CI = 4.4–5.5). The primary ICU admission diagnoses documented by physicians and nurses were respiratory distress in 19 (54%) patients and sepsis in 16 (45.7%). Admission APACHE score was a significant predictor of mortality (18.5 ± 1.3 for those who survived to discharge and 23.4 ± 1.4 died prior to discharge; *p* = 0.038). Fifteen of the 35 were intubated, 19 received pressor therapy, and four received hemodialysis in the ICU. Of the 15 patients intubated, 8 (53%) did not survive to discharge. Seven of the 8 who did not survive to discharge had acute respiratory distress; one was postoperative. Among the 19 who received pressor therapy, 13 died (68%). Among the 4 (11%) who underwent hemodialysis while in the ICU, only one survived to discharge. Ten received both pressor therapy and endotracheal intubation. The mortality in this subgroup of patients was 70% (Table 2). Mean ICU length of stay for patients who survived to discharge was 4.2 days (CI = 2.7–5.7) and for patients who died in hospital was 3 days (CI = 1.5–4.5). Eleven patients (31%, 95% CI: 24–28%) had palliative care consults and 5 (14%; 95% CI: 8.2–19.8%) saw a chaplain. Of the patients who survived, 9 were discharged to their home, 5 were discharged to hospice, and 4 were discharged to a post-acute care facility. In total, 25 of the 35 (71%; 95% CI: 63.3–78.7%) DNR patients who were admitted to the ICU died or could not care for themselves in the home environment.

## 4. Limitations

This is a retrospective chart review study. We were dependent on nursing and physician documentation in the EHR, which may not have been complete or comprehensive. The study was performed at a single medical center. Patient, family, physician, and nursing culture may play a significant role in clinical decision making. In our cohort, we do not present data on comorbidity other than the APACHE II score, which is likely an important determinant of median to long-term survival of patients with critical illness with and without DNR status [4]. As all patients had DNR status prior to admission to an ICU, our reported cohort does not include patients admitted to an ICU after major elective or nonemergency surgery, and hence our results are not applicable to patients with DNR status who require ICU admission after such events.

## 5. Conclusions/Discussion

With the aging of the population and the increased availability of life prolonging medical technology, end of life care has become an important aspect of all medial practice [5]. Regulatory agencies encourage or mandate that providers ask patients to affirm their wishes concerning their end of life care [6]. There is insufficient data about the factors that impact DNR and end of life decision making.

Health care spending and more efficient use of resources are also becoming an increasingly prominent part of medical practice, and there is insufficient evidence to guide the use of intensive interventions as treatments for patients who choose to limit life sustaining technologies [1, 5]. This study reports the outcomes of DNR patients who were admitted to an intensive care unit in a single medical center during an 18-month period. These patients represent a small percentage (0.48%) of the total patients admitted during the study period. They had a high mortality. Sixteen (45.7%) did not survive to hospital discharge, and those who were discharged had a high likelihood of being transferred to a hospice or post-acute care facility. These results suggest the importance of regular and repeated discussions with patients and families about their wishes regarding end of life care when diagnosis is a terminal or critical illness. Our patients were infrequently consulted to palliative care and chaplain assistance, despite the fact that these interventions have been determined to have significant value to patients and their families at end of life [7, 8]. This single site, retrospective study highlights the need for further investigation into the efficacy of ICU admission for DNR patients as well as the need for advancing the evidence based practice of end of life care.

## Figures and Tables

**Table 1:** Demographics and outcome of patients who were DNR and then admitted to an ICU.

	Discharged alive (*n* = 19)	Expired (*n* = 16)	*p* values
Age	78.4 ± 2.6	82.4 ± 2.6	0.35
Sex (M/F)	9/10	6/10	0.57
Apache score	18.5 ± 1.3	23.4 ± 1.7	0.038
Serum creatinine	1.66 ± 0.26	1.5 ± 0.2	0.67
Serum lactate	2.88 ± 1.0	2.80 ± 0.4	0.93
Intubated	7/19	8/16	0.44
Use of pressor therapy	6/19	13/16	0.003
ICU length of stay	4.2	3	0.25
Palliative consult	5/19	6/16	0.49
Days in ICU prior to palliative consult	1.8 ± 0.7	1.5 ± 0.6	0.88
Chaplain	1/19	4/16	0.1

**Table 2:** ICU care and outcome of patients who were DNR prior to admitting to an ICU.

	Number of patients	Percentage expired
DNR prior to ICU	35	46
Intubated	15/35	53
Hemodialysis	4/35	75
Received pressor therapy	19/35	68
Both intubated and received pressor therapy	10/35	70
